# Decreased prevalence of cancer in patients with multiple sclerosis: A case-control study

**DOI:** 10.1371/journal.pone.0188120

**Published:** 2017-11-27

**Authors:** Xavier Moisset, Maud Perié, Bruno Pereira, Emilie Dumont, Christine Lebrun-Frenay, François-Xavier Lesage, Frederic Dutheil, Frederic Taithe, Pierre Clavelou

**Affiliations:** 1 Université Clermont-Auvergne, INSERM, Neuro-Dol, CHU Clermont-Ferrand, Service de Neurologie Clermont-Ferrand, France; 2 CHU Clermont-Ferrand, Service de Neurologie, Clermont-Ferrand, France; 3 CHU Clermont-Ferrand, Délégation Recherche Clinique & Innovation, Clermont-Ferrand, France; 4 CHU Pasteur 2, Service de Neurologie, Nice, France; 5 Univ Paul Valéry Montpellier 3, Univ. Montpellier, EPSYLON EA, CHU Montpellier, service sante au travail et pathologie professionnelle, Montpellier, France; 6 Université Clermont Auvergne, CNRS, LaPSCo, Stress physiologique et psychosocial, CHU Clermont-Ferrand, Service Santé Travail Environnement, WittyFit, Clermont-Ferrand, France; 7 Australian Catholic University, Faculty of Health, Melbourne, Victoria, Australia; University of Pennsylvania, UNITED STATES

## Abstract

**Background:**

Studies of cancer prevalence have produced conflicting results concerning the relative risk of overall and specific sub-types of cancer in patients with multiple sclerosis (MS). Contemporary controls and information on tobacco use and alcohol consumption are generally missing from previous studies.

**Objectives:**

To evaluate lifetime cancer prevalence in a large cohort of MS patients relative to appropriate controls.

**Methods:**

We conducted a case-control study, using a postal survey of a cohort of MS patients. Of the 1574 questionnaires sent, 1107 could be used for statistical analysis. Data from 1568 controls were prospectively collected using the same self-administered survey among consecutive out-patients in a single neurology department. Propensity scores matched on age, gender, and history of smoking and alcohol consumption were calculated.

**Results:**

Among the MS patients, 7.32% had ever presented with a cancer, whereas 12,63% of the controls had, leading to a bootstrap matched odds ratio (OR) of 0.63; 95% CI 0.57–0.70. Although only exploratory, the use of DMT (immunomodulators or immunosupressants) did not appear to increase this risk (p = 0.42). The disease course also did not affect cancer prevalence.

**Conclusion:**

MS was associated with a reduced overall cancer risk.

## Introduction

The immune system plays an important role in both multiple sclerosis (MS) [1] and cancer [2], making it plausible that cancer risk is modified by auto-immune diseases such as MS. Some authors have hypothesized that some immunological characteristics of MS disease activity improve antitumor surveillance [3]. Others have proposed that disease modifying treatments (DMT), especially immunosuppressive treatments (IS), could increase cancer incidence in these patients [4,5]. Studies of cancer incidence and prevalence have produced conflicting results concerning the relative risk of overall and specific sub-types of cancer in MS patients, showing either a decreased or increased risk relative to the general population [6,7]. These studies all have several weaknesses such as the lack of contemporary controls, the absence of information concerning the two major cancer risk factors, tobacco use and alcohol consumption, the potential role of DMTs, and the probable underestimation of cancer prevalence due to incomplete reporting. Indeed, a recent meta-analysis calculated a global estimate of overall cancer prevalence in MS of 4.39% that seems to be very low [6].

The primary objective of the present study was to evaluate lifetime cancer prevalence in a large cohort of MS patients relative to contemporary controls coming from the same geographical area and matched for age, gender, history of tobacco use and alcohol consumption. The secondary exploratory objectives were to assess the potential impact of DMTs on overall cancer risk and to determine whether cancer prevalence was different between patients with different disease courses.

## Materials and methods

### Subjects

The survey was conducted among the members of the “MS patients’ network in Auvergne”, an administrative area in the centre of France. Patients can join this association only if their MS diagnosis has been made by a neurologist whether they are followed in a hospital or by a private neurologist. Patients with Clinically Isolated Syndrome (CIS) have been able to join the association since 2013. A database was set-up with demographic and medical information concerning the association members. According to the MS prevalence in Auvergne (1800 in 2013), this database contained 87% of MS patients from our catchment area (1,360,000 inhabitants in 2013). The patients association database is approved by the National Commission of Data Processing and Civil Liberties (CNIL), in accordance with French law. Controls were outpatients visiting the Neurology Department of Clermont-Ferrand University Hospital and their accompanying persons. The current study was approved by the appropriate Institutional Review Board (Comité de Protection des Personnes Sud-Est 06, 2014/CE28).

### Survey questionnaire

A self-administered questionnaire was sent to all 1574 members of the network in March 2014. The inclusion of consecutive outpatients seen in the Neurology department of the university hospital and their accompanying person was carried out between August 2014 and August 2015. This control population was selected as it was coming from the same catchment area as MS patients and was not receiving any immunomodulatory or immunosuppressant treatments for chronic diseases. Moreover, there are no local or national cancer registries and when available, such registries never contain informations about risk factors. Questionnaires were secondarily anonymized and only one person (MP) had access to the corresponding list.

Both MS patients and controls had to respond to questions concerning their gender, date of birth, history of cancer lesion, smoking history (daily use during at least one year), and history of alcohol consumption (daily consumption during at least one year). We chose to be very sensitive as even a low dose can promote cancer development. Patients with a history of cancer also had to note the year of diagnosis, the implicated organ, and the name of their oncologist or general practitioner to allow retrieval of the pathological confirmation of the cancer. To limit the risk of cancer under-reporting, the question was “Have you ever had a cancer or a cancerous lesion (in particular of the skin, cervix or cancerous intestinal polyp)?”. This question was followed by a list of 22 potential cancer locations to make the reporting easier. MS patients also had to include data concerning their inflammatory disease: type of disease (MS or CIS), evolutive course of MS (relapsing-remitting (RR), secondary progressive (SP), primary progressive (PP)), date of first MS signs, and name of DMT taken during at least three months. Information declared by patients concerning MS was confirmed using the clinical database of our hospital and the local European Database for Multiple Sclerosis [8] by the only person who had access to the identity of the participants. People of the control group had only to state the reason for their consultation or to note that they were an accompanying person. This information was confirmed during the face to face interview. Face validity was tested on 10 healthy volunteers and 10 patients. No other validation was performed on this simple home-made questionnaire. For MS patients receiving the questionnaire and its accompanying letter, they were free to participate or not to this survey. For these patients, sending the questionnaire back was considered as a written consent to participate. Informed verbal consent was obtained for every single person completing the questionnaire in the Neurology Department.

Patients consulting for a cancer-related neurological problem (mainly primary or secondary brain lesion and chemotherapy-induced peripheral neuropathy) were excluded.

The type of DMT was divided into four groups to allow statistical analysis: patients who had received immunomodulatory therapies alone (IM), IS alone, both IM and IS, and those who had never received any treatment. In the analyses of the impact of DMT, only cancers that occurred after the first symptoms of MS, i.e. after the potential introduction of a DMT, were taken into account.

CIS, RR-MS and SP-MS were grouped for the analysis of disease course, as they correspond to three stages of the same form of the disease. PP-MS represented the other group.

### Sample size calculation

To evaluate lifetime cancer prevalence in a large cohort of MS patients, the sample size estimation was focused principally on the precision of the associated 95% confidence interval due to the lack of precise data about the overall cancer prevalence in MS patients. Inclusion of more than 900 MS patients in the study allows a maximum margin of error of ±2% for a cancer prevalence of between 5% and 15%. To evaluate the prevalence of MS patients relative to controls, the statistical power to highlight an odds-ratio (OR) around 0.65, with a two-sided type I-error α of 5%, seemed satisfactory (80%) with more than 900 subjects in each group (MS patients and controls) [9].

### Statistical analysis

Statistical analysis was performed using Stata software (StataCorp LP, College Station, US). The tests were two-sided, with α = 0.05. Subject characteristics are presented as the mean ± standard-error of the mean or median [interquartile range] for continuous data (assumption of normality assessed using the Shapiro–Wilk test). Comparisons between groups were performed using chi-squared or Fisher’s exact tests for categorical variables, and the Student t-test or Mann-Whitney test for quantitative parameters (normality, homoscedasticity studied using the Fisher-Snedecor test). Multivariate analysis was performed using generalized linear model (with logit link function for dichotomous dependent outcome). The covariates were retained according to univariate results and to their clinical relevance. The covariates retained for multivariate analyses performed to estimate the ORs for the impact of DMTs and impact of MS disease course were also gender, age, alcohol and tobacco consumption. A particular focus was considered for the study of interactions between factors and multicollinearity. Results were expresses as odds-ratios (OR) and 95% confidence interval. As the baseline characteristics of MS patients differ from control subjects, propensity score matching, defined by Rosenbaum and Rubin (1983), was performed. The estimated propensity score is the predicted probability of group (MS or controls), derived from the fitted regression model (logistic regression). As described by Austin [10], “stratification on the propensity score involves stratifying subjects into mutually exclusive subsets based on their estimated propensity score. Subjects are ranked according to their estimated propensity score. Subjects are then stratified into subsets based on previously defined thresholds of the estimated propensity score.” Therefore, when the propensity score has been correctly specified, the distribution of measured baseline covariates will be approximately similar between cases and controls within the same stratum. The covariates included in the propensity score model were known to be clinically relevant and fixed according to univariate results: gender, age, alcohol and tobacco consumption. As described for multivariate analyses, a particular attention was paid to interactions and multicollinearity. Although there are different approaches to matching, the most common approach in the medical literature is nearest neighbor pair matching without replacement within specified calipers of the propensity score [11]. So, matchit command from MatchIt package (R software) has been used to perform this analysis with a caliper width of 0.2. Then, according to several recommendations, we have proposed an approach based on bootstrap, a well-known resampling method for estimating the standard error of estimated statistics and of constructing confidence intervals [12]. After bootstrap simulations (1000 simulations), the odds ratio (OR) estimated for each randomly chosen group of patients were calculated and are presented with the 95% confidence interval. As described by several authors [13], assessment of residual confounding using sample-size independent tests, such as standardized differences, has been proposed to evaluate the quality of treatment bias matching.

## Results

### Populations

Overall, 1111 out of the 1574 eligible MS patients responded to the survey, i.e. 70.6% of our cohort. There was no difference in the average age of the responders and non-responders (50.8 ± 0.4 vs 51.6 ± 0.6, p = 0.25), but the proportion of females was greater among responders (79.2% vs 73.7%, p = 0.016). At the same time, 1590 consecutive non-MS outpatients or their accompanying person were also included. These control subjects were not-included if they were consulting for a problem related to a malignancy (for example epilepsia due to a cancer lesion or chemotherapy-induced neuropathy). Among the 2701 subjects of the study, 26 (1%) were not included in the analyses concerning demographic features, risk factors, and cancer prevalence because of missing data or consultation due to a cancer. Finally, these analyses were performed on data from 1107 MS patients and 1568 controls. MS patients were younger, more likely to be female, and had a more frequent history of tobacco use. Alcohol consumption was more frequent in non-MS patients (**Table 1**). Among the MS patients, 829 (75%) had a CIS, RR-MS or SP-MS and 76 (7%) had PP-MS. Information concerning the diagnosis was missing for 202 patients (18%).

**Table 1:** Comparison of demographic characteristics and risks factors for cancer among patients with MS (n = 1107) and controls (n = 1568). SEM: Standard error of the mean; MS: Multiple sclerosis.

	Patients with MS (n = 1107)	Controls (n = 1575)	P
**Age: mean ± SEM**	50.7 ± 0.4	56.7 ± 0.5	<0.001
**Female gender: % (n)**	79.3 (878)	53.2 (834)	<0.001
**History of tobacco smoking: % (n)**	49.8 (551)	42.8 (671)	<0.001
**History of alcohol drinking: % (n)**	10.7 (118)	18.8 (294)	<0.001

### Overall lifetime cancer prevalence and the prevalence for specific subtypes

In the MS group, 81 patients had had 84 cancers, three patients having had two, for an overall cancer prevalence of 7.32%. The most frequent cancers were breast and skin cancers including basal-cell carcinomas. In the control group, 198 subjects had had 214 cancers, 16 subjects having had two for an overall cancer prevalence of 12,63%. The most frequent cancers were urological (with a majority being prostate cancer), breast, and skin cancers (**Table 2**). 11 patients had declared a cancer that was not confirmed by pathology (5 in the MS group, 6 in the control group).

**Table 2:** Overall lifetime cancer prevalence and specific subtypes of cancer prevalence in patients with MS (n = 1107) and controls (n = 1568).

	Patients with MS % (n)	Controls % (n)
**Patients with history of cancer**	7.32 (81 patients, 84 cancers)	12.63 (198 patients, 214 cancers)
**Breast cancers**	2.53 (28)	2.42 (38)
**Skin cancers** ▪ **Basal-cell carcinomas** ▪ **Squamous-cell carcinomas** ▪ **Melanomas** ▪ **Others skin cancers**	1.36 (15) ▪ 0.90 (10) ▪ 0.00 (0) ▪ 0.36 (4) ▪ 0.09 (1)	2.04 (32) ▪ 1.21 (19) ▪ 0.06 (1) ▪ 0.64 (10) ▪ 0.13 (2)
**Urological cancers** ▪ **Prostatic cancers** ▪ **Renal cancers** ▪ **Bladder cancers** ▪ **Testicular cancers**	0.72 (8) ▪ 0.27 (3) ▪ 0.36 (4) ▪ 0.09 (1) ▪ 0.00 (0)	2.93 (46) ▪ 1.98 (31) ▪ 0.57 (9) ▪ 0.32 (5) ▪ 0.06 (1)
**Gynecological cancers other than breast** ▪ **Ovarian cancers** ▪ **Uterine cancers (cervical or endometrial)**	1.17 (13) ▪ 0.09 (1) ▪ 1.08 (12)	1.79 (28) ▪ 0.45 (7) ▪ 1.34 (21)
**Gastro-intestinal cancers** ▪ **Colorectal cancers** ▪ **Oesophageal cancers** ▪ **Gastric cancers** ▪ **Others GI cancers**	0.90 (10) ▪ 0.81 (9) ▪ 0.00 (0) ▪ 0.09 (1) ▪ 0.00 (0)	1.53 (24) ▪ 1.28 (20) ▪ 0.06 (1) ▪ 0.00 (0) ▪ 0.19 (3)
**Brain tumors**	0.00 (0)	0.13 (2)
**Thyroid cancers**	0.36 (4)	0.57 (9)
**Hemopathies**	0.36 (4)	1.02 (16)
**Naso-pharyngeal cancers**	0.18 (2)	0.57 (9)
**Bronchopulmonary cancers**	0.00 (0)	0.64 (10)

### Overall cancer prevalence after propensity score analysis and bootstrap

**Fig 1:**
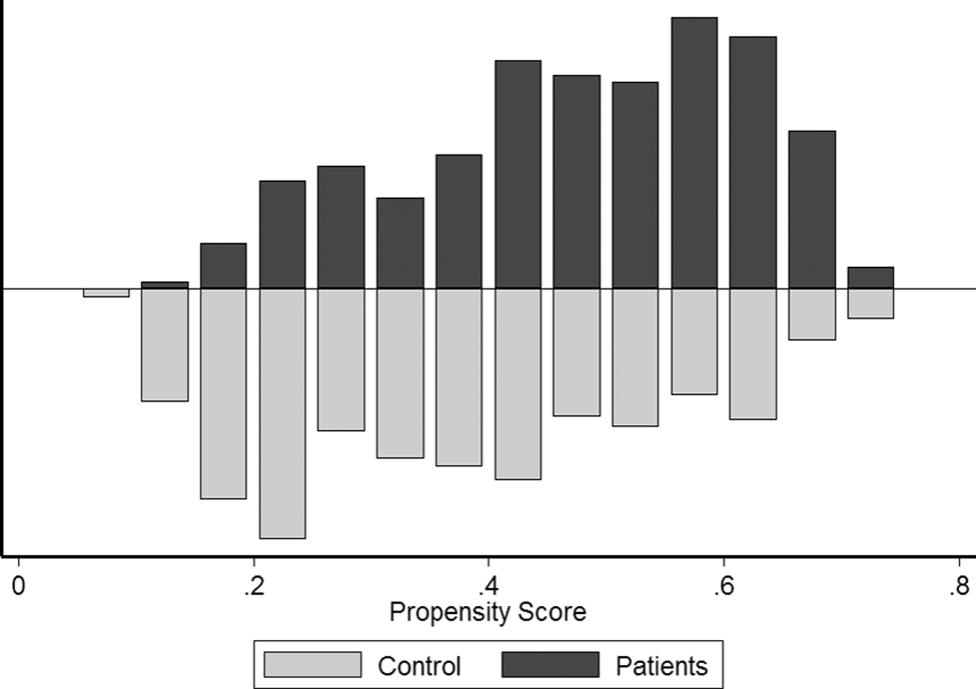
Propensity score distribution in MS patients and controls. Patients and controls were matched using a propensity score with nearest neighbor pair matching without replacement within specified calipers (width of 0.2). 1000 bootstrap simulations were then performed.

**Fig 2:**
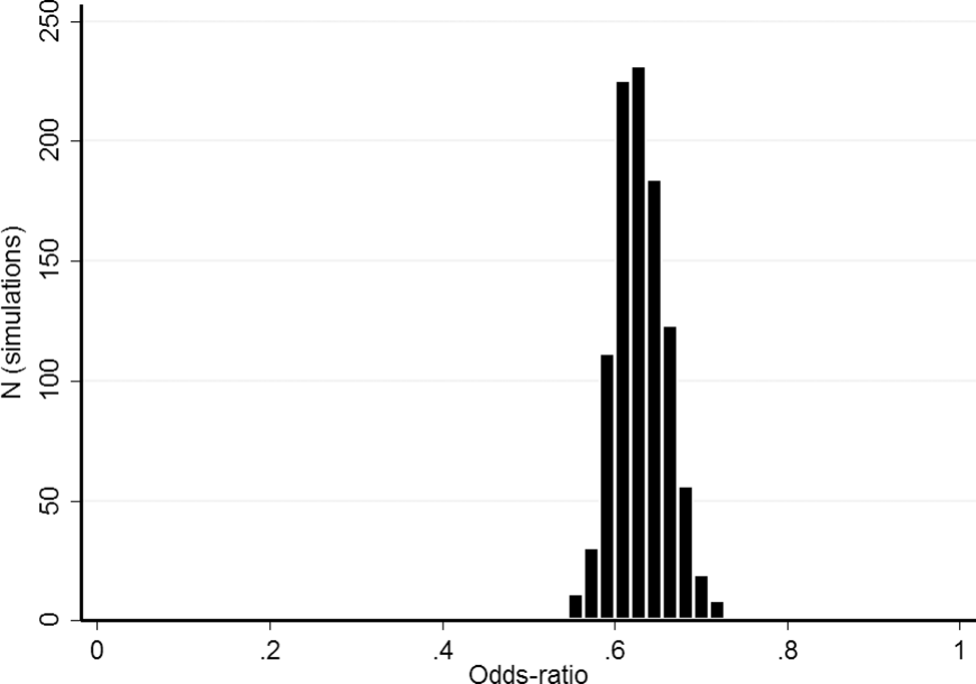
Odds ratio estimates using bootstrap simulations (1000 randomly chosen group of patients based on the propensity score model).

The propensity score matched on age, gender, and history of smoking and alcohol consumption (**Fig 1**), allowing us to match 993 MS patients with 993 non-MS subjects, was performed 1000 times. The standardized difference for the four covariates was not significantly different between MS patients and controls (**S1 Fig**). The mean OR was 0.63 (95% CI = 0.57–0.70) for overall cancer prevalence **(Fig 2**).

### Impact of DMTs

Data concerning treatments were available for 1089 MS patients (98.0%). The DMTs received during at least three months are listed in **Table 3**. Among the MS patients, 61 had a history of cancer after the first symptoms of MS (5.60%). Characteristics of the four treatment sub-groups are presented in **Table 4.** Using multivariate analysis, patients who received treatment had no greater risk of cancer than those who did not receive treatment (OR 1.22; 95% CI 0.63–2.35; p = 0.55 for IM; OR 0.82; 95% CI 0.33–2.06; p = 0.67 for IS; and OR 0.78; 95% CI 0.33–1.80; p = 0.55 for patients who had received at least one IM and one IS treatment).

**Table 3:** Disease modifying treatments received for at least 3 months. IM: immunomodulatory disease modifying treatments; IS: Immunosuppressant disease modifying treatments. The total is above 100% as many patients have received more than one DMT during the time course of their disease.

	% (n)
**IM** ▪ **Interferon beta** ▪ **Glatiramer Acetate** ▪ **Dimethyl fumarate**	64.6 (703) ▪ 52.5 (572) ▪ 23.0 (250) ▪ 0.2 (2)
**IS** ▪ **Fingolimod** ▪ **Natalizumab** ▪ **Mitoxantrone** ▪ **Methotrexate** ▪ **Azathioprine** ▪ **Cyclophosphamide** ▪ **Mycophenolate Mofetil** ▪ **Teriflunomide** ▪ **Rituximab**	31.8 (346) ▪ 4.3 (47) ▪ 10.2 (111) ▪ 4.0 (44) ▪ 9.6 (104) ▪ 5.1 (55) ▪ 4.3 (47) ▪ 3.4 (37) ▪ 0.1 (1) ▪ 0.1 (1)
**None**	24.8 (270)

**Table 4:** Demographic characteristics, risk factors and overall cancer prevalence in different clusters of DMT. SEM: Standard error of the mean. IM: immunomodulatory disease modifying treatments; IS: Immunosuppressant disease modifying treatments.

	IM only (n = 465)	IS only (n = 114)	IM and IS (n = 229)	None (n = 264)	p
**Age: mean ± SEM**	47.1 ± 0.5	56.5 ± 1.2	49.0 ± 0.8	55.6 ± 0.8	<0.001
**Female gender: % (n)**	78.7 (366)	75.4 (86)	80.8 (185)	79.6 (210)	0.71
**History of tobacco smoking: % (n)**	53.6 (249)	38.6 (44)	52.8 (121)	46.6 (123)	0.02
**History of alcohol consumption: % (n)**	10.3 (48)	7.9 (9)	7.9 (18)	14.39 (38)	0.08
**Overall cancer prevalence: % (n)**	5.59 (26)	6.14 (7)	3.93 (9)	7.20 (19)	0.48

### Impact of MS disease course

The CIS, RR and SP-MS population was younger and with a higher proportion of women than the PP-MS population. Tobacco use did not differ between the two sub-groups but alcohol consumption was more frequent in the PP-MS group **(Table 5).** Multivariate analysis did not find differences in cancer prevalence based on the course of the disease (OR 1.11; 95% CI 0.45–2.76; p = 0.818).

**Table 5:** Demographic characteristics, risk factors and overall cancer prevalence according to MS disease course. PP: Primary Progressive; RR: Recurring Remitting; SP: Secondary Progressive; SEM: Standard error of the mean.

	PP-MS (n = 76)	CIS, RR-MS and SP-MS (n = 829)	p
**Age: mean ± SEM**	57.8 ± 1.2	49.4 ± 0.4	<0.001
**Female gender: % (n)**	64.5 (49)	80.5 (667)	0.001
**History of tobacco smoking: % (n)**	40.8 (31)	51.3 (425)	0.08
**History of alcohol consumption: % (n)**	18.4 (14)	9.9 (82)	0.02
**Overall cancer prevalence: % (n)**	7.89 (6)	6.51 (54)	0.63

## Discussion

This study assessed the life-time prevalence of cancer in MS patients and contemporary controls, taking into account tobacco use and alcohol consumption. Our results show that there was a decreased prevalence of overall cancer risk in patients with MS (OR: 0.63; 95 CI 0.57–0.70) relative to matched subjects, despite use of DMTs.

Several previous studies have found a similar decreased cancer risk, in accordance with our results. Kingwell and collaborators found a standardized incidence ratio (SIR) of cancer of 0.86 (95 CI 0.78–0.94) in a cohort study conducted in more than 6000 Canadian MS patients [14]. Similar results have been obtained in France in a multicenter survey of 7418 MS patients (SIR: 0.29; CI 95% 0.17–0.45 in men and 0.53; CI 95% 0,42–0,66 in women)[15]. This was confirmed in a recent meta-analysis [6]. However, other authors have reported no difference or an increased risk of cancer prevalence in MS patients relative to the general population [6,7].

We present, for the first time, a prevalence estimate based on a large cohort of MS patients compared to a contemporary control group matched for age, gender, tobacco use and alcohol consumption, together with the impact of DMTs. Unlike previous studies based on cancer registries as a control group, we investigated the cancer risk in patients from the same geographic area and have taken into account all cancer types, especially non-melanoma skin cancers that were often excluded in registries. This under-reporting is highlighted by the results of a recent meta-analysis that found an overall cancer prevalence in MS of 4.4%, far below the 7.3% reported in the present study [6]. Another strength of our survey is that it is the first to take into account two major risk factors for cancer (tobacco use and alcohol consumption).

Based on a recent meta-analysis, the risk of urinary system cancers appeared to be higher than expected, whereas the risk of pancreatic, ovarian, prostate, and testicular cancer were lower than expected [6]. In our cohort, we found the most frequent cancer type in MS patients to be breast cancer, which would be expected in a population of middle-aged women. Evaluation of the risk for each type of cancer was not possible in this study because of an insufficient number of cancers of each type to allow sufficient statistical power for such an analysis.

We found no increased risk of cancer in patients who had received DMT, whether it was IM or IS. Our results are in accordance with current knowledge concerning classical IM [4,16]. Fewer studies have focused on the impact of IS on cancer risk in MS patients, as anti-cancer treatments are well-known for inducing secondary neoplasms. Some studies suggest an increased cancer risk for patients who received azathioprine, cyclophosphamide or several IS [4], whereas another did not find any increased risk in patients receiving cyclophosphamide [17]. Another study has recently shown that the overall incidence of malignancies was only slightly increased in patients that had received mitoxantrone, but the risk of leukemia and colorectal cancer was higher [5]. Notwithstanding, the cancer risk of such treatments is well-known for indications in other inflammatory diseases [5,18]. Rheumatoid arthritis patients receiving methotrexate have an increased risk of lymphoma, but it seems that this risk is linked to the disease and not its treatment [19].

Anti-tumor immunosurveillance may provide the physiological explanation for the reduced cancer-risk in MS patients [20,21]. Indeed, autoimmunity is a form of hyper-vigilance against self-antigens, and may be one of the mechanisms leading to the development of MS. Further studies are required to address this issue, for example, those investigating the properties of the lymphocytes of MS-patients. It is noteworthy that several DMTs are also used, or are being evaluated, for their potential anti-tumor activity. Examples include several DMTs such as dimethyl fumarate, that induces necroptosis in colon cancer cells [22], fingolimod, that is being tested on various cancers to increase the efficacy of other drugs, [23] and teriflunomide, that has been shown to have anticancer activity against triple negative breast cancer cells [24]. Mitoxantrone is a well-known anti-neoplastic drug. Thus, DMTs may inhibit cancer development in some cases, although they are used differently in oncology.

One limitation of our study stems from the restricted area in which it was conducted, leading to a number of cancer cases that is too small to obtain conclusions for each cancer sub-type. Moreover, self-reporting of cancer can be seen as a bias, although the conditions to look for cancer were the same in both patients and controls. Another limitation is that the study was not powered to detect differences in cancer rates across DMT subgroups. Thus, although these differences are non-significant, we cannot draw any firm conclusions. Moreover, the relative timing for DMT treatment and cancer is not precisely known. Careful monitoring of the cancer risk related to these treatments will be still needed in the coming years, especially for the newer ones. Data concerning the duration of DMT exposure are also lacking and must be taken into account in future studies, as the cancer risk could be dose-dependent. A final limitation concerns cancer risk factors. There is a lack of information concerning the intensity and duration of alcohol consumption and tobacco use. Indeed, we had only binary data for the presence or absence of daily alcohol consumption or tobacco use for at least one year during the patients’ life. The impact of dose is well-known for these two factors and should have been evaluated. Other risk factors should be explored in future studies (estrogen use, UV exposure, nutritional habits, body mass index, viral infections,…).

In conclusion, MS was associated with a reduced overall cancer risk. Classical immunomodulator (beta-interferons and glatiramer acetate) and immunosuppressant use was not associated with an increased overall cancer risk but there is not sufficient data to draw firm conclusions. Concerning all these drugs and especially the newest, data from larger cohorts and after longer exposure times is mandatory.

## Supporting information

S1 Table Reason for consultation of controls subjects. (DOCX)

S1 Fig Standardized difference for the 4 covariates (age, gender, history of smoking and alcohol consumption) included in the 1000 bootstrap simulations. Using propensity score matching, the distribution of observed baseline covariates (age, gender, and history of smoking and alcohol consumption) was similar between controls and MS patients.
(TIFF)

S1 File Competing_Interests_Statement. (DOCX)

S2 File Ethics_statement. (DOCX)

S3 File Survey questionnaire for patients with multiple sclerosis. Original version written in French.
(DOCX)

S4 File Survey questionnaire for patients with multiple sclerosis. Translated version written in English.
(DOCX)

S5 File Survey questionnaire for controls. Original version written in French.
(DOCX)

S6 File Survey questionnaire for controls. Translated version written in English.
(DOCX)
