# Supplementary material for: Decreased prevalence of cancer in patients with multiple sclerosis: A case-control study
Source: PLoS One. 2017 Nov 27;12(11):e0188120. doi: 10.1371/journal.pone.0188120 (PMC5703510; doi:10.1371/journal.pone.0188120)
Supplement: S1 Table — (DOCX) [file pone.0188120.s001.docx]

| Reason for consultation | % (n) |
| --- | --- |
| Neuromuscular disease | 9,89 (155) |
| Movement Disorder | 15,18 (238) |
| Neurovascular disease | 14,41 (226) |
| Epilepsy | 12,37 (194) |
| Sleep disorder | 10,65 (167) |
| Headache | 4,85 (76) |
| Cognitive decline | 1,59 (25) |
| Motorneuron disease | 1,53 (24) |
| Functional complaint | 2,30 (36) |
| Others | 6,76 (106) |
| Accompanying person | 20,47 (321) |

**Reason for consultation of controls subjects.**
