# Supplementary figures and images for: Decreased prevalence of cancer in patients with multiple sclerosis: A case-control study
Source: PLoS One. 2017 Nov 27;12(11):e0188120. doi: 10.1371/journal.pone.0188120 (PMC5703510; doi:10.1371/journal.pone.0188120)

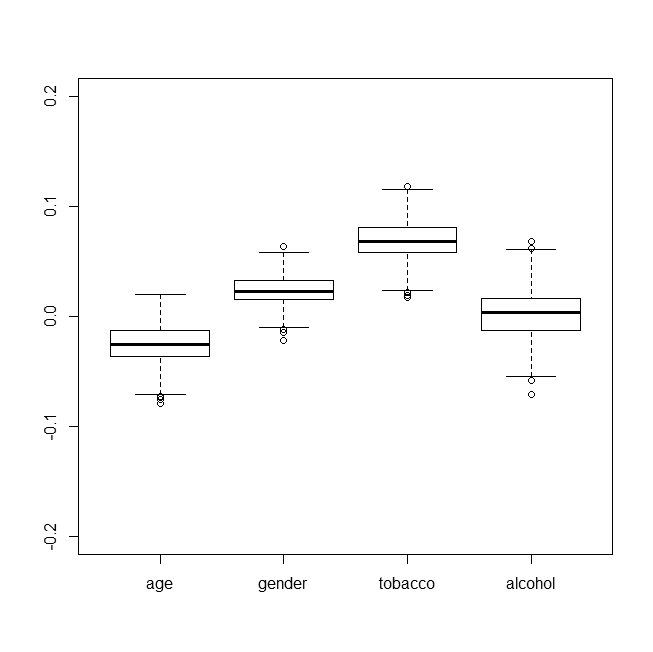

Supplement: S1 Fig — Using propensity score matching, the distribution of observed baseline covariates (age, gender, and history of smoking and alcohol consumption) was similar between controls and MS patients. (TIFF) [file pone.0188120.s002.tiff]
