# Supplementary material for: Decreased prevalence of cancer in patients with multiple sclerosis: A case-control study
Source: PLoS One. 2017 Nov 27;12(11):e0188120. doi: 10.1371/journal.pone.0188120 (PMC5703510; doi:10.1371/journal.pone.0188120)
Supplement: S1 File — (DOCX) [file pone.0188120.s003.docx]

Competing Interests Statement

XM reports non-financial support from Merck-Serono, Biogen, Sanofi-Pasteur-MSD, and Genzyme, personal fees from Astellas and from Institut UPSA de la douleur, not relating to the submitted work; FT reports non-financial support from Bayer, LFB, Novartis, Merck-Serono, Biogen, and Genzyme, personal fees from Biogen, not relating to the submitted work; PC reports personal fees from Teva-Pharma, Merck-Serono, Novartis, Biogen, Genzyme, Bayer, and Almirall, not relating to the submitted work. CLB reports non financial supports from Biogen, Bayer, Genzyme, Merck Serono and Teva. MP reports non financial support from Lundbeck, Teva and Biogen. This does not alter our adherence to PLOS ONE policies on sharing data and materials.

We received funding from “TEVA pharma”. These funding was used to print questionnaires, and to buy stamps and envelopes to send them. This does not alter our adherence to PLOS ONE policies on sharing data and materials.

Yours sincerely,

Xavier Moisset and Maud Perie, on behalf of the authors.

**Corresponding author:**

Xavier MOISSET

Neurology Department, Clermont-Ferrand University Hospital

F-63000 Clermont-Ferrand, FRANCE

Tel: +33 4 73 752 200; Fax: +33 4 73 752 202

E-mail: xavier.moisset@gmail.com
