# Supplementary material for: Decreased prevalence of cancer in patients with multiple sclerosis: A case-control study
Source: PLoS One. 2017 Nov 27;12(11):e0188120. doi: 10.1371/journal.pone.0188120 (PMC5703510; doi:10.1371/journal.pone.0188120)
Supplement: S2 File — (DOCX) [file pone.0188120.s004.docx]

Ethics statement

The current study was approved by the appropriate Institutional Review Board (Comité de Protection des Personnes Sud-Est 06, 2014/CE28).

We received funding from “TEVA pharma”. This funding was used to print questionnaires, and to buy stamps and envelopes to send them. The funders had no role in study design, data collection and analysis, decision to publish, or preparation of the manuscript.

Yours sincerely,

Xavier Moisset and Maud Perie, on behalf of the authors.
