# Supplementary material for: Decreased prevalence of cancer in patients with multiple sclerosis: A case-control study
Source: PLoS One. 2017 Nov 27;12(11):e0188120. doi: 10.1371/journal.pone.0188120 (PMC5703510; doi:10.1371/journal.pone.0188120)
Supplement: S3 File — Original version written in French. (DOCX) [file pone.0188120.s005.docx]

Madame, Monsieur,

Par ce présent courrier, nous souhaiterions vous solliciter pour un travail de recherche clinique, en tant que membre du réseau SEP Auvergne.

De rares études ont été réalisées dans le monde sur le risque d’avoir un cancer lorsque l’on a une SEP (ou une pathologie apparentée). En effet, certains craignaient que l’anomalie du système immunitaire à l’origine de la SEP puisse favoriser l’émergence de cancer. De plus, les traitements de fond de la SEP ont également un impact sur le système immunitaire et pourraient favoriser le développement de certains cancers. Heureusement, il n’en est rien, puisque les quelques études réalisées jusqu’à présent indiquent au contraire une légère **diminution du risque d’avoir un cancer**. Nous souhaitons confirmer cela dans une population de taille importante. Il vous suffira de remplir un bref questionnaire, afin de vérifier ce possible effet protecteur.

Les questionnaires seront traités de façon anonymisée. Des données médicales (forme de votre maladie, durée, traitement) seront prises en compte. Comme pour toute base de données, vous possédez un droit d'accès, de rectification et d'opposition à ces données.

**Merci de prendre quelques minutes pour remplir ces feuilles et nous les retourner grâce à l’enveloppe-réponse ci-jointe. Même si vous n’avez jamais eu de cancer ou de lésion cancéreuse, votre réponse est très importante pour que les résultats de cette étude soient fiables ! Les résultats de cette étude seront présentés lors de la prochaine réunion du réseau SEP (SEPthon), prévue à l’automne. Il est toutefois essentiel que nous puissions avoir votre réponse avant la fin du mois de juin.**

En vous remerciant d’avance pour votre participation,

Dr M. LAUXEROIS Pr P. CLAVELOU

(Président du Conseil d’Administration) (Président du comité de pilotage)

Numéro d’anonymat : ….

**Date de naissance : … / … / ……**

**Sexe : Homme** ❒ **Femme** ❒

**Avez-vous eu au cours de votre vie un cancer ou une lésion cancéreuse (notamment de la peau, du col de l’utérus ou un polype cancéreux du colon)?**

**OUI**  ❒ **NON**  ❒

**1 - Habitudes de vie :**

Etes-vous fumeur(se) ou avez-vous fumé tous les jours pendant plus d’un an au cours de votre vie ?

**OUI**  ❒ **NON**  ❒

Consommez-vous tous les jours de l’alcool (vins, bière, cidre, apéritifs, …) ou avez-vous consommé de l’alcool tous les jours pendant plus d’un an au cours de votre vie ?

**OUI**  ❒ **NON**  ❒

**Si vous n’avez jamais eu de cancer, passez à la question 4**

**2 - Année de diagnostic du cancer (ou de la lésion cancéreuse) :**

**3 - Localisation de la lésion cancéreuse (entourez la réponse) :**

- Sein
- Colon / Rectum
- Poumon
- Prostate
- ORL (Lèvre / Bouche / Pharynx / Larynx)
- Peau : Mélanome, carcinome épidermoïde ou basocellulaire
- Ovaire
- Utérus / Col de l’utérus
- Vessie
- Sang : Leucémie, Lymphome
- Pancréas
- Rein
- Autre : ……..

**Pour confirmer la nature exacte de la lésion cancéreuse, nous avons besoin de contacter le médecin qui vous a pris en charge (médecin spécialiste, cancérologue ou à défaut médecin traitant).**

Nom du médecin : ………………………………………….

Ville d’exercice : …………………………………………………………………

**4 - Informations concernant votre maladie inflammatoire :**

Diagnostic (entourez la réponse correspondant à votre situation):

- SEP
- Premier événement démyélinisant (Clinically Isolated Syndrome)
- Neuromyélite optique (Maladie de Devic)
- Je ne sais pas

**Si vous avez une SEP, votre forme de maladie est :**

- Rémittente (RR)
- Progressive : progressive primaire (PP) ou secondairement progressive (SP)
- Je ne sais pas

**Date des premiers signes de la maladie inflammatoire (mois/année) : ……………..**

**Traitement(s) de fond déjà utilisé(s),** pour une durée d’au moins 3 mois durant l’ensemble de votre prise en charge**:**

- Aucun
- Avonex
- Bétaféron
- Cellcept (Mycophénolate mofétil)
- Copaxone
- Extavia
- Gilenya
- Imurel (azathioprine)
- Méthotrexate
- Mitoxantrone (Novantrone, Elsep)
- Rebif
- Tysabri
- Protocole de recherche : ……………………
- Autre : ………………………..
