# Supplementary material for: Decreased prevalence of cancer in patients with multiple sclerosis: A case-control study
Source: PLoS One. 2017 Nov 27;12(11):e0188120. doi: 10.1371/journal.pone.0188120 (PMC5703510; doi:10.1371/journal.pone.0188120)
Supplement: S4 File — Translated version written in English. (DOCX) [file pone.0188120.s006.docx]

Dear Mr, Dear Mrs,

Because you are member of the MS patients’ network in Auvergne, we ask you to participate to a survey about cancer prevalence in patients with multiple sclerosis (MS).

Few surveys have been performed into the world about cancer risk in patients with multiple sclerosis (or other inflammatory diseases or the central nervous system). Indeed, multiple sclerosis is caused by a dysfunction of the immune system and some medical authors wonder if this dysfunction could promote the development of cancer. Moreover, disease modifying therapies, used as treatments of multiple sclerosis, have an impact on the immune system and could also increase cancer risk. Fortunately, this seems not to be the case, since different surveys seems to indicate at the opposite a slight **decrease in cancer risk among MS patients.** Our study aims to confirm this result in a large population. To participate, you have to complete a brief questionnaire.

Questionnaires will be anonymised. Medical informations (diagnosis, disease duration, treatments) will be taken into account. As in all databases, you have the right to access the data, to modify them and a right of opposition to this data.

**Thank you for taking the time to complete this questionnaire and to return it thanks to the pre-paid return envelope attached. Even if you have never had cancer, your answer is important. Results of this survey will be exposed during a next meeting of MS patients’ network.**

Thank you in advance for your participation

Dr M. LAUXEROIS Pr P. CLAVELOU

(President of the board of directors) (President of the steering committee)

Anonymity number: ….

Date of birth: … / … / ……

Sex : Male ❒ Female ❒

**Have you ever had, during your lifetime, a cancer or a cancerous lesion (including skin cancer, cervical cancer or cancerous colic polyp)?**

**YES**  ❒ **NO**  ❒

**1 -Lifestyles:**

Do you smoke or have you ever smoked daily during at least one year during your life?

**YES**  ❒ **NO**  ❒

Do you consume alcohol daily (wine, beer, cider, appetizers…) or have you ever consumed alcohol daily during at least one year during your life?

**YES**  ❒ **NO**  ❒

**2 – Year of cancer’s diagnosis: ……….**

**3 - Localisation of cancerous’ lesion :**

- Breast
- Colorectal
- Lung
- Prostate
- ENT (Lips / Mouth / Pharynx / Larynx)
- Skin : Malignant melanoma, Spinal carcinoma, Basocellular carcinoma
- Ovary
- Uterine cervix
- Bladder
- Blood : Leukemia, Lymphoma
- Pancreas
- Kidney
- Other : ………………..

**In order to confirm the exact nature of your lesion, we need to contact the doctor who managed your cancer (specialist) or your general practitioner.**

Name of the doctor : ………………………………………….

City : ……………………………………………

**4 – Informations about your inflammatory disease:**

Diagnosis

- Multiple sclerosis
- Clinically Isolated Syndrome
- Neuromyelitis optica
- I don’t know

**If you suffer from multiple sclerosis, what is the evolutive course of the disease :**

- Relapsing Remitting (RR)
- Progressive : primary progressive (PP) ou secondary progressive (SP)
- I don’t know

**Date of first symptoms of the disease (month/year) : ……………..**

**Disease modifying therapies you have ever received during at least 3 months :**

- None
- Avonex
- Bétaféron
- Cellcept (Mycophénolate mofétil)
- Copaxone
- Extavia
- Gilenya
- Imurel (azathioprine)
- Méthotrexate
- Mitoxantrone (Novantrone, Elsep)
- Rebif
- Tysabri
- Research protocol : ……………………
- Other : ………………………..
