# Supplementary material for: Decreased prevalence of cancer in patients with multiple sclerosis: A case-control study
Source: PLoS One. 2017 Nov 27;12(11):e0188120. doi: 10.1371/journal.pone.0188120 (PMC5703510; doi:10.1371/journal.pone.0188120)
Supplement: S5 File — Original version written in French. (DOCX) [file pone.0188120.s007.docx]

Nom :……………………………Prénom : …………………………

Pathologie :………………………………………

Date de naissance : … / … / ……

Sexe : Homme ❒ Femme ❒

**Avez-vous eu au cours de votre vie un cancer ou une lésion cancéreuse (notamment de la peau, du col de l’utérus ou un polype cancéreux du colon)?**

**OUI**  ❒ **NON**  ❒

**1 - Habitudes de vie :**

Etes-vous fumeur(se) ou avez-vous fumé tous les jours pendant plus d’un an au cours de votre vie ?

**OUI**  ❒ **NON**  ❒

Consommez-vous tous les jours de l’alcool (vins, bière, cidre, apéritifs, …) ou avez-vous consommé de l’alcool tous les jours pendant plus d’un an au cours de votre vie ?

**OUI**  ❒ **NON**  ❒

**2 - Année de diagnostic du cancer (ou de la lésion cancéreuse) : ……….**

**3 - Localisation de la lésion cancéreuse (entourez la réponse) :**

- Sein - Colon / Rectum
- Poumon - Prostate
- ORL (Lèvre / Bouche / Pharynx / Larynx)
- Peau : Mélanome, carcinome épidermoïde, carcinome basocellulaire
- Ovaire - Utérus / Col de l’utérus
- Vessie - Sang : Leucémie, Lymphome
- Pancréas - Rein
- Autre : ………………..

**Pour confirmer la nature exacte de la lésion cancéreuse, nous avons besoin de contacter le médecin qui vous a pris en charge (médecin spécialiste, cancérologue ou à défaut médecin traitant).**

Nom du médecin : ………………………………………….

Ville d’exercice : ……………………………………………
