# Supplementary material for: Decreased prevalence of cancer in patients with multiple sclerosis: A case-control study
Source: PLoS One. 2017 Nov 27;12(11):e0188120. doi: 10.1371/journal.pone.0188120 (PMC5703510; doi:10.1371/journal.pone.0188120)
Supplement: S6 File — Translated version written in English. (DOCX) [file pone.0188120.s008.docx]

Surname :……………………………First name : …………………………

Reason for consultation or hospitalization :………………………………………

Date of birth: … / … / ……

Sex : Male ❒ Female ❒

**Have you ever had, during your lifetime, a cancer or a cancerous lesion (including skin cancer, cervical cancer or cancerous colic polyp)?**

**YES**  ❒ **NO**  ❒

**1 -Lifestyles:**

Do you smoke or have you ever smoked daily during at least one year during your life?

**YES**  ❒ **NO**  ❒

Do you consume alcohol daily (wine, beer, cider, apéritifs …) or have you ever consumed alcohol daily during at least one year during your life?

**YES**  ❒ **NO**  ❒

**2 – Year of cancer’s diagnosis: ……….**

**3 - Localisation of cancerous lesion :**

- Breast
- Colorectal
- Lung
- Prostate
- ENT (Lips / Mouth / Pharynx / Larynx)
- Skin : Malignant melanoma, Spinal carcinoma, Basocellular carcinoma
- Ovary
- Uterine cervix
- Bladder
- Blood : Leukemia, Lymphoma
- Pancreas
- Kidney
- Other : ………………..

**In order to confirm the exact nature of your lesion, we need to contact the doctor who managed your cancer (specialist) or your general practitioner.**

Name of the doctor : ………………………………………….

City : ……………………………………………
